# pKalculator: A p*K*_a_ predictor for C–H bonds

**DOI:** 10.3762/bjoc.20.144

**Published:** 2024-07-16

**Authors:** Rasmus M Borup, Nicolai Ree, Jan H Jensen

**Affiliations:** 1 Department of Chemistry, University of Copenhagen, Copenhagen, DK-2100, Denmark

**Keywords:** C–H p*K*_a_ values, p*K*a predictor

## Abstract

Determining the p*K*_a_ values of various C–H sites in organic molecules offers valuable insights for synthetic chemists in predicting reaction sites. As molecular complexity increases, this task becomes more challenging. This paper introduces pKalculator, a quantum chemistry (QM)-based workflow for automatic computations of C–H p*K*_a_ values, which is used to generate a training dataset for a machine learning (ML) model. The QM workflow is benchmarked against 695 experimentally determined C–H p*K*_a_ values in DMSO. The ML model is trained on a diverse dataset of 775 molecules with 3910 C–H sites. Our ML model predicts C–H p*K*_a_ values with a mean absolute error (MAE) and a root mean squared error (RMSE) of 1.24 and 2.15 p*K*_a_ units, respectively. Furthermore, we employ our model on 1043 p*K*_a_-dependent reactions (aldol, Claisen, and Michael) and successfully indicate the reaction sites with a Matthew’s correlation coefficient (MCC) of 0.82.

## Introduction

Over the years, the ability to selectively break a C–H bond to create new connections has attracted increasing interest [1]. While past methods allowed for C–H transformations in simple molecules, recent synthetic protocols [2] enable selective C–H activation and diversification in larger molecules. This has, for example, attracted the pharmaceutical industry to implement such C–H transformations to diversify different types of molecules ranging from small drug-like molecules to intermediates and lead compounds. Especially late-stage functionalization is a promising emerging field that allows chemists to efficiently explore the chemical space in complex molecules by exchanging a C–H bond with different functional groups to modify the biological activity of drugs [2]. However, pinpointing which C–H bond is reacting can be challenging.

Grzybowski and co-workers recently addressed this gap by predicting p*K*_a_ values for C–H bonds in dimethyl sulfoxide (DMSO) using a graph convolutional neural network (GCNN) [3]. Using a mix of experimental and computed p*K*_a_ data, they achieved a mean absolute error (MAE) of 2.1 p*K*_a_ units. Lee and co-workers also addressed this problem by creating a general machine learning (ML) model using either a neural network or XGBoost. They trained on experimental p*K*_a_ values in 39 solvents from the “internet Bond-energy Databank” (iBonD). Thus, they could predict the lowest p*K*_a_ value for a wide range of molecules that contain bonds such as N–H, O–H, C–H, S–H, and P–H. However, they reported a scarcity of non-aqueous p*K*_a_ values and achieved a MAE of 1.5 p*K*_a_ units for the solvent DMSO using XGBoost [4–5]. Unfortunately, neither the Grzybowski group nor the Lee group have made their models generally available to other users.

Inspired by the efforts of the Grzybowski group and the Lee group, we have developed pKalculator, a quantum chemistry (QM)-based workflow for the automatic computation of C–H p*K*_a_ values in DMSO. The computed C–H p*K*_a_ values are then used to generate training data for an ML model using LightGBM [6]. The QM-based workflow and the ML model are freely available under the MIT license.

## Methods

### Datasets

We compile a dataset of 732 experimental p*K*_a_ values in DMSO from two different sources, Bordwell [7] and iBonD [4]. The Bordwell dataset contains experimental C–H p*K*_a_ values in DMSO from 419 molecules. For the iBonD database, we select experimental C–H p*K*_a_ values in DMSO for 313 molecules. As the iBonD database only contains an image of each molecule, we employ the “Deep Learning for Chemical Image Recognition” software (DECIMER v. 2.0), developed by Rajan and co-workers [8–10]. While DECIMER converts molecular images into SMILES, manual intervention is required to ensure the SMILES string correctly represents the molecule. Finally, to mirror the dataset by Roszak et al. [3], we also incorporate 43 heterocycles without experimental p*K*_a_ values from Shen et al., leaving us with a dataset of 775 compounds [11]. This dataset will be used to calculate QM p*K*_a_ values using our QM workflow described in the next section.

We also create a dataset from Reaxys that contains 1043 p*K*_a_-controlled reactions. These reactions include 584 aldol, 408 Claisen, and 51 Michael reactions. This dataset is used as an out-of-sample dataset to see how well our ML model predicts the reaction site. Additionally, we use six pharmaceutical intermediates that undergo selective borylation to compare our QM workflow and ML model with experimentally determined reaction sites.

### The quantum chemistry-based workflow

Following work by Ree et al. [12–15], we present a fully automated QM-based workflow for computing C–H p*K*_a_ values. A given SMILES string undergoes modifications to produce a list of SMILES for each deprotonated C–H bond. We generate min(1 + 3*n*_rot_, 20) conformers for each SMILES using RDKit (v.2022.09.4) [16–17], where (*n*_rot_) represents the number of rotatable bonds. Each conformer undergoes optimization in dimethyl sulfoxide (DMSO, ε = 47.2) using GFN-FF-xTB [18] and the analytical linearized Poisson–Boltzmann (ALPB) equation [19] as the implicit solvation model. We then remove conformers with relative energies above 3 kcal/mol and select unique conformers by taking the centroids of a Butina clustering using pairwise heavy-atom root mean square deviation (RMSD) with a threshold of 0.5 Å [16,20]. For more information, refer to Supporting Information File 1, section “Selecting unique conformers”.

Subsequently, we re-optimize the remaining conformers in DMSO with GFN2-xTB [21] and the ALPB implicit solvation model to identify the lowest-energy conformer. We then conduct re-optimization in ORCA (v. 5.0.4) [22–23], using the dispersion D4-corrected DFT functional CAM-B3LYP [24–25], the Karlsruhe [26–27] triple-ζ basis set, def2-TZVPPD, and the conductor-like polarizable continuum model (CPCM) [28] as the implicit solvation models. CAM-B3LYP is chosen as the optimal functional based on a benchmark study that evaluates the accuracy of different levels of theory, ranging from semiempirical methods (xTB) [21] over composite electronic structure methods (r^2^SCAN-3c) [29] to DFT methods (CAM-B3LYP) [24–25]. All these methods are evaluated as single-point calculations or optimization and frequency calculations. For comprehensive details, refer to Supporting Information File 1, section “Benchmark study - computational methods”. Hereafter, we check the geometries for imaginary frequencies and use the total thermal energy at 298.15 K. Following the approach of the Grzybowski group [3], we compute the heterolytic dissociation energy through the direct deprotonation reaction, 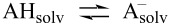; see Equation 1.


[1] $$\Delta G{}^{\circ}=E\left({\text{A}}_{\text{solv}}^{-}\right)-E\left({\text{AH}}_{\text{solv}}\right).$$

For each set of deprotonated C–H sites in a molecule, we determine the minimum heterolytic dissociation energy (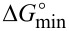). Hereafter, we assume a linear relationship between the experimental p*K*_a_ values and 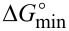 as this assumption allows us to derive the empirical constants *a* and *b* and correct any systematic errors; see Equation 2, where Δ*G*° is replaced by 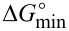. After retrieving the empirical constants *a* and *b*, we can determine the QM-computed p*K*_a_ values for all deprotonated C–H sites using Equation 2:


[2] $$\text{p}K_{\text{a}}=a\cdot \Delta G{}^{\circ}+b.$$

### Machine learning

#### The feature descriptor

Recent research shows that the atomic descriptors introduced by Finkelmann et al. [30–31], using charge model 5 (CM5) atomic charges [32], are a great representation of atoms in molecules that can be used in combination with an ML model to predict a variety of properties. These properties encompass the site of metabolism [31,33], the strengths of hydrogen bond donors and acceptors [34–36], and the regioselectivity of electrophilic aromatic substitution reactions [14]. Building on the methodology from Finkelmann et al. [30–31] and Ree et al. [14], we utilize the automated approach to compute CM5 atomic charges from semiempirical tight-binding (GFN1-xTB [37]) calculations. We modify the workflow to enhance the accuracy of the computed CM5 atomic charges. Instead of generating a single random conformer, we produce 20 random conformers from a SMILES string and optimize the structure with molecular mechanics force fields [38] using RDKit [16]. The CM5 atomic charges of the lowest-energy conformer are then used to generate atomic descriptors based on sorting the CM5 charges for a given atom of the input SMILES string. Furthermore, we adjust the shell radius from 5 to 6, improving the performance of the ML model to predict p*K*_a_ values as detailed in Supporting Information File 1, section “The descriptor”.

#### Data preparation and hyperparameter optimization

Building on the procedure outlined by Ree et al. [14], we employ the Optuna framework (v. 3.3.0) [39] to identify optimal hyperparameters for LigthGBM regression and classification models [6]. Specifically, the Bayesian optimization technique utilizing the tree-structured Parzen estimator is applied for hyperparameter space exploration. For the regression task, the target value are the QM-computed p*K*_a_ values. For the binary classification task, which aims to predict the site with the lowest QM-computed p*K*_a_ value, labels are assigned in the following manner: ‘1’ for the lowest QM-computed p*K*_a_ value (true site) and ‘0’ for all other QM-computed p*K*_a_ values. As there is sometimes a slight variation between the p*K*_a_ value and the other p*K*_a_ values, we also introduce a tolerance where a p*K*_a_ value within +1 p*K*_a_ units or +2 p*K*_a_ units of the lowest p*K*_a_ value is accepted as ‘1’ to account for these variations, see Supporting Information File 1, section “Machine learning models” for more information. Further, given the significant imbalance between the two classes (with ‘0’s far outnumbering ‘1’s), the hyperparameter *scale_pos_weight* is invoked during hyperparameter optimization. Finally, we establish a “null model” for the classification task, wherein all sites are predicted as ‘0’.

The dataset with QM-computed p*K*_a_ values (775 compounds; 3910 p*K*_a_ values) is initially split randomly by compound into a training set (80%; 620 compounds; 3121 p*K*_a_ values) and a held-out test set (20%; 155 compounds; 789 p*K*_a_ values). For each ML model, we carry out a fivefold randomly shuffled cross-validation. Within each fold, the original training set is further split randomly into a new training set (90% of the original training set) and a validation set (10% of the original training set). This allows us to evaluate different models and estimate their performance. Hereafter, each ML model is trained on our original training set and tested against the held-out test set. Finally, we select the best-performing ML model.

## Results and Discussion

### Computing p*K*_a_ values

**Figure 1:**
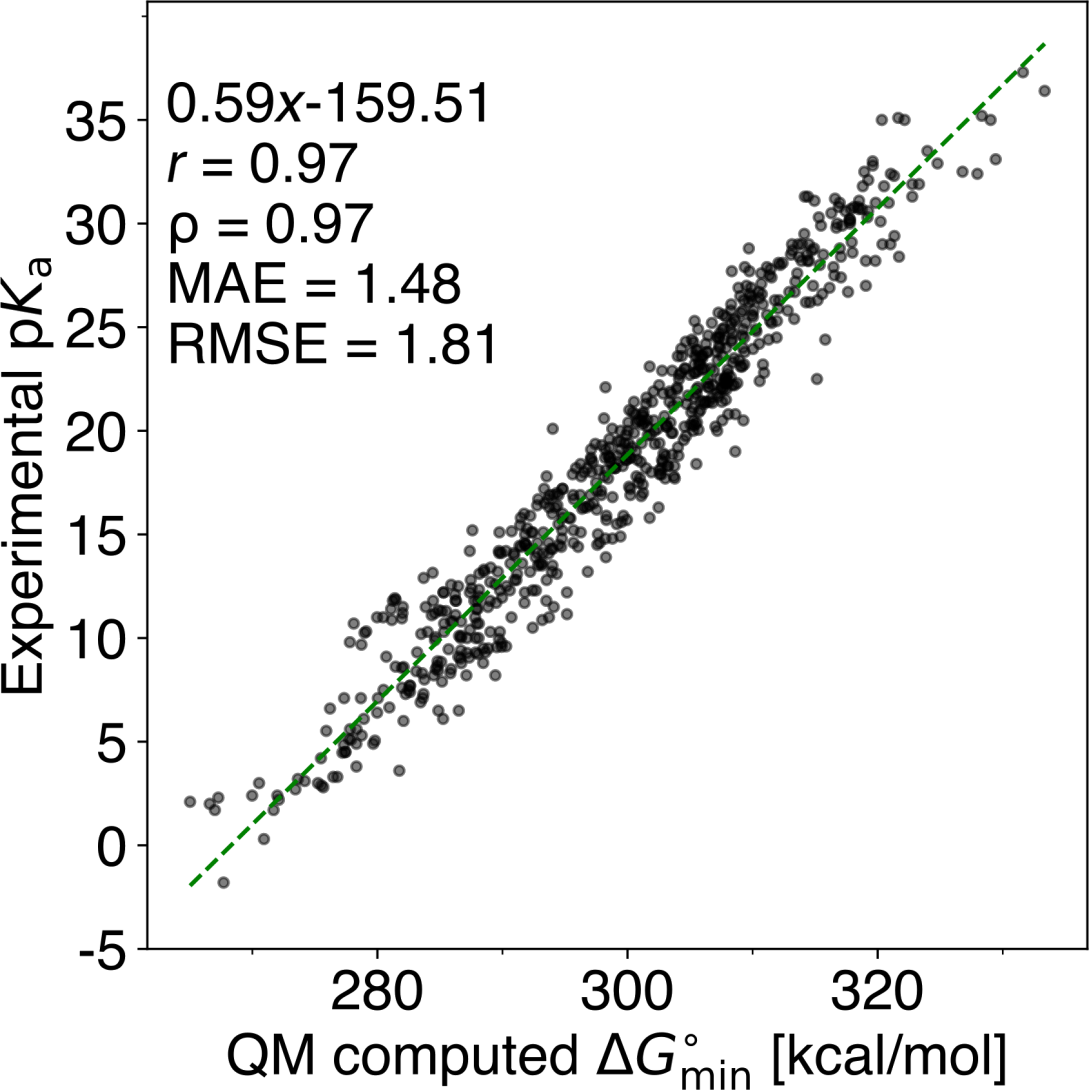
Correlating computed 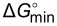 values and experimental p*K*_a_ values for 695 compounds. *r*: Pearson correlation coefficient; ρ: Spearman’s rank correlation coefficient; MAE: mean absolute error; RMSE: root mean squared error. QM calculations were carried out at the CAM-B3LYP/def2-TZVPPD CPCM(DMSO)//GFN2-xTB ALPB(DMSO) level of theory.

From section “The quantum chemistry-based workflow” above, we can determine the empirical values *a* and *b* in Equation 2. For each set of deprotonated sites in a molecule, we extract the computed 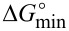 value and fit it against the experimental p*K*_a_ values. Hereafter, we convert the computed 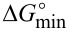 to QM-computed p*K*_a_ values using Equation 2. We then inspect outliers that exceed an absolute p*K*_a_ unit difference of 5 p*K*_a_ units between the experimental p*K*_a_ value and the QM-computed p*K*_a_ value. We choose an absolute p*K*_a_ unit difference of 5 p*K*_a_ units to ensure that the QM-computed p*K*_a_ is well above the error that is to be expected on the level of theory we are using (CAM-B3LYP). The observed outliers typically result from one of the following reasons: (i) calculation errors concerning the expected minimum p*K*_a_ site, (ii) discrepancies between literature structures and database structures, (iii) mislabeled experimental p*K*_a_ values, or (iv) extrapolated p*K*_a_ values. Notably, the extrapolated p*K*_a_ values correspond to compounds beyond the scale measurable in DMSO (p*K*_a_ ≥ 35) because of the autoprotolysis of DMSO (p*K*_a_(DMSO) = 35) [40–41]. For more information regarding finding and removing outliers, see Supporting Information File 1, section “Finding outliers”. After multiple iterations, we identified 695 molecules to have reliable experimental p*K*_a_ values and computed 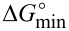 values. The values for the computed 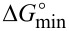 are then fitted against the experimental p*K*_a_ values, leaving us with empirical constants *a* and *b*; see Figure 1. We now use the derived linear regression to convert all computed Δ*G*° values into QM-computed p*K*_a_ values for our whole dataset (775 compounds). These values are used as target values for the ML part.

### Machine learning models for predicting C–H p*K*_a_ values

**Figure 2:**
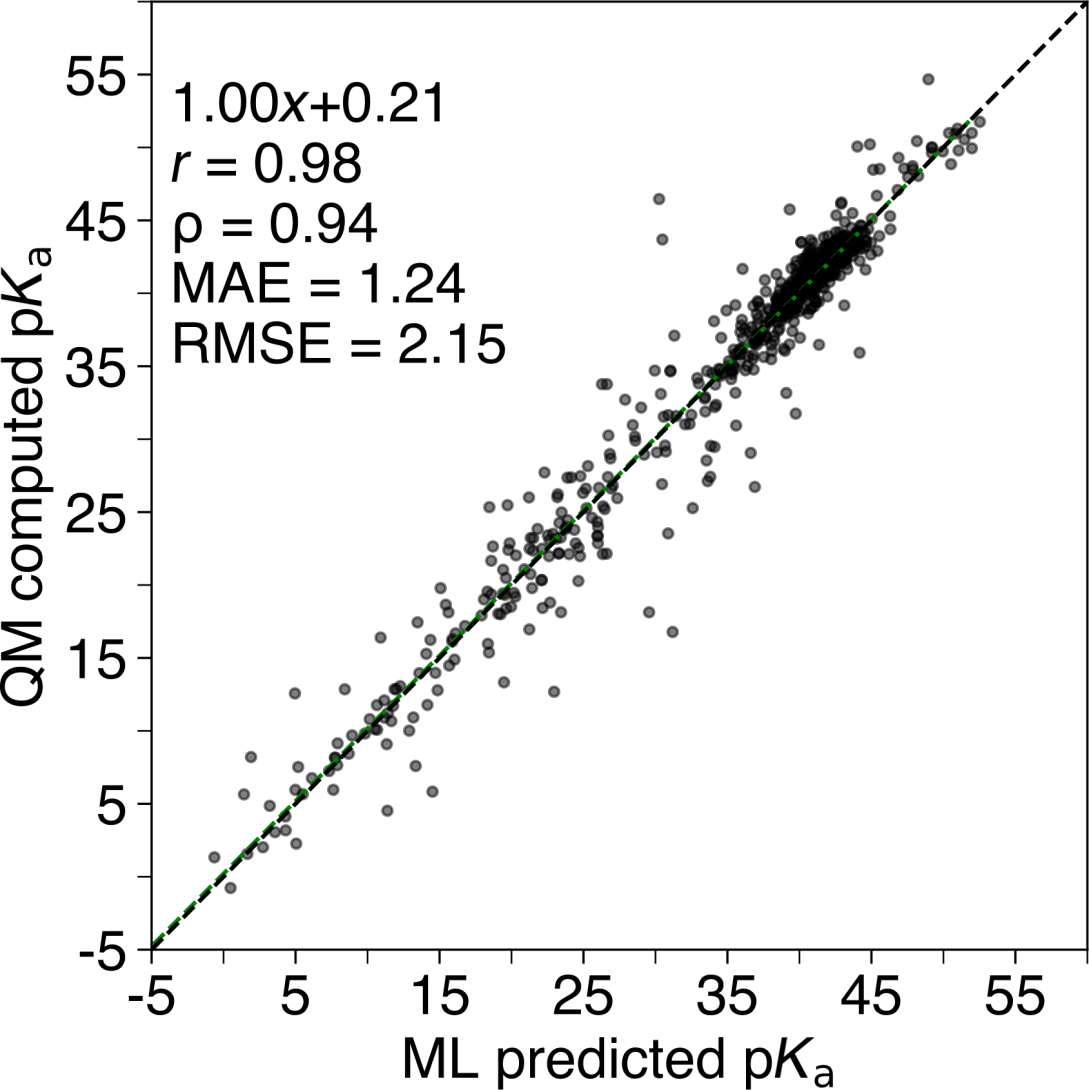
ML-predicted p*K*_a_ values vs QM-computed p*K*_a_ values of the held-out test set (155 compounds; 789 p*K*_a_ values). *r*: Pearson correlation coefficient; ρ: Spearman’s rank correlation coefficient; MAE: mean absolute error; RMSE: root mean squared error. All predictions were made using the best ligthGBM regressor. All calculations were carried out at the CAM-B3LYP/def2-TZVPPD CPCM(DMSO)//GFN2-xTB ALPB(DMSO) level of theory.

To learn and predict C–H p*K*_a_ values, we train a LightGBM regression model with our generated dataset containing QM-computed p*K*_a_ values (775 compounds; 3910 p*K*_a_ values). Hereafter, we correlate and compare the ML-predicted p*K*_a_ values and the QM-computed p*K*_a_ values and achieve a MAE and a RMSE of 1.24 and 2.15 p*K*_a_ units, respectively, for the held-out test set (155 compounds; 789 p*K*_a_ values), as illustrated in Figure 2. When zooming in on the ML-predicted p*K*_a_ values that are not correlating well with the QM-computed p*K*_a_ values, we find C–H sites that are either bridgeheads or where the negative charge is stabilized by resonance. This may be due to the nature of the chosen descriptor vector based on sorted CM5 atomic charges as it may not take into account, for example, steric strain and charge delocalisation. We discuss this further in Supporting Information File 1, section “Outliers for the test set”.

We then compare our ML model with previously reported ML models for predicting p*K*_a_ values, namely, the GCNN C–H p*K*_a_ predictor by Roszak et al. [3] and the XGBoost p*K*_a_ predictor by Yang et al. [5]. Roszak et al. [3] used a mix of experimental data (414 compounds) [7], manually curated DFT data (212 compounds), and previously reported DFT data (194 C–H sites) [11]; they obtained a MAE of 2.18 p*K*_a_ units for their test set. Yang et al. [5] used filtered entries from the iBonD dataset, comprising 15338 compounds and 19397 p*K*_a_ values across 39 solvents [5]. As they not only predict C–H p*K*_a_ values, we cannot compare our result with their best ML model. However, they also report a holistic six-solvent (HM-6S) XGBoost model in DMSO (9.3% of the data), which most likely contains the majority of C–H p*K*_a_ values. For this XGBoost model, they achieved MAE and RMSE values of 1.53 and 2.35 p*K*_a_ units, respectively. A comparison between our ML model, the GCNN model of Roszak et al., and the model of Yang et al. is shown in Table 1. While a direct comparison with these studies is not feasible because of differing datasets, our model surpasses Roszak et al.’s GCNN model by a MAE of 0.94 p*K*_a_ units and outperforms Yang et al.’s HM-6S model by a MAE of 0.29 p*K*_a_ units.

**Table 1:** Comparing different ML models for predicting p*K*_a_ values. Mean absolute error (MAE) and root mean squared error (RMSE) are provided in p*K*_a_ units.

Method	MAE	RMSE

**LGBM (this work)**	**1.24**	**2.15**
GCNN [3]	2.18	—
XGBoost HM-6S (DMSO)^a^ [5]	1.53	2.35

^a^HM-6S: Table 7 in their paper.

### Predicting the lowest C–H p*K*_a_ value

Now that we can fairly accurately predict p*K*_a_ values with our LightGBM regressor, another use case is to be able to identify the C–H site with the lowest p*K*_a_ value to predict the site of reaction. For this purpose, we treat the task as a binary classification and train both a LightGBM classifier and a LightGMB regressor. As described earlier in section “Data preparation and hyperparameter optimization”, the QM-computed p*K*_a_ values are translated into binary values, with ‘1’ representing the lowest QM-computed p*K*_a_ value and ‘0’ representing other QM-computed p*K*_a_ values. The performance metrics for the test set demonstrate that the regression model (MCC of 0.97) outperforms the classification model (MCC of 0.92) when used as a binary classifier, as seen in Table 2.

**Table 2:** Test set performance metrics: comparison between a LightGBM classifier and a LightGBM regressor for binary classification of the lowest p*K*_a_ site. Reaxys performance metrics: comparison between a LightGBM classifier and a LightGBM regressor for binary classification of the reaction site in Reaxys. The best model is marked in bold.^a^

	Test set performance metrics						Reaxys performance metrics					
method	ACC	MCC	PPV	TPR	TNR	NPV	ACC	MCC	PPV	TPR	TNR	NPV

null model^b^	0.80	0	0	0	1.00	0.80	0.87	0	0	0	1.00	0.87
classifier	0.97	0.92	0.97	0.90	0.99	0.98	0.92	0.70	0.64	0.85	0.93	0.98
**regressor**	**0.99**	**0.97**	**0.97**	**0.98**	**0.99**	**1.00**	**0.96**	**0.82**	**0.84**	**0.84**	**0.98**	**0.98**

^a^ACC: accuracy; MCC: Matthew's correlation coefficient; PPV: precision/positive predictive value; TPR: recall/true-positive rate; TNR: specificity/true-negative rate; NPV: negative predictive value. ^b^All predicted p*K*_a_ values are “0” to highlight the imbalance of the dataset.

Now we train a LightGBM classifier and a LightGMB regressor for the entire dataset (775 compounds; 3910 pK_a_ values) of QM-computed p*K*_a_ values to assess the generalization capability of our ML models. We use an out-of-sample dataset of 1043 p*K*_a_-dependent reactions from Reaxys, containing 584 aldol, 408 Claisen, and 51 Michael reactions. These reactions are chosen because they all involve a deprotonation step, and the C–H site with the lowest p*K*_a_ value is most likely the site of the reaction. We also use these reactions for comparison with Roszak et al. [3], who evaluated their GCNN model against 12873 p*K*_a_-controlled reactions, including aldol, Claisen and Michael reactions, and correctly predicted the reacting site with an accuracy of 90.5%. Our out-of-sample set is also used to see how well our ML models predict the site of reaction using the lowest ML-predicted p*K*_a_ value.

**Scheme 1:**
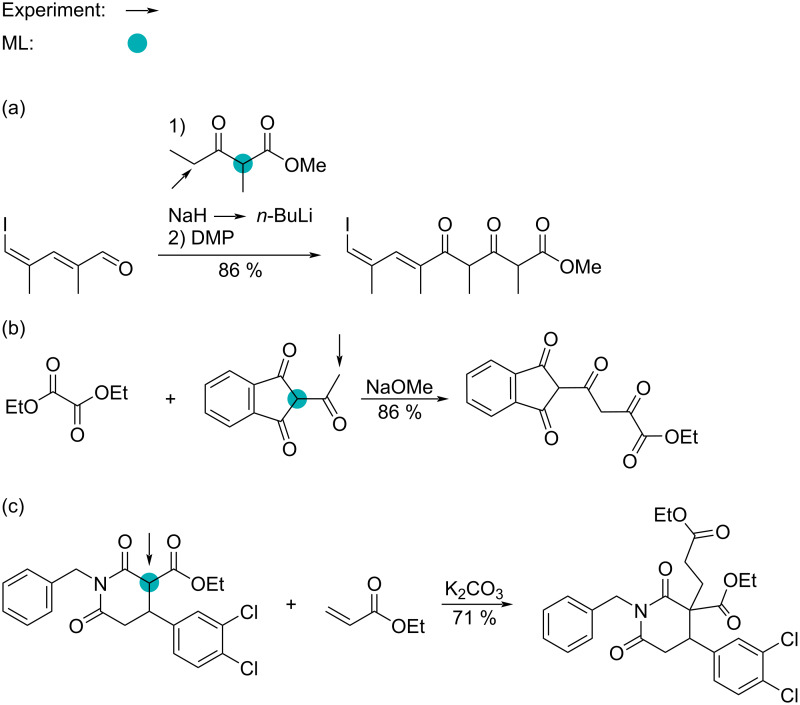
Predicting the reaction site for three different reactions from the out-of-sample dataset from Reaxys. (a) Aldol reaction, Reaxys reaction ID: 9947221 [42]; (b) Claisen reaction, Reaxys reaction ID: 3402137 [43]; (c) Michael reaction, Reaxys reaction ID: 29819768 [44]. Arrow: experimental site; teal filled circle: ML-predicted lowest p*K*_a_.

To understand the result for the out-of-sample set, we show three different reactions in Scheme 1. The first step of the reaction shown in Scheme 1a is an aldol reaction where the deprotonation occurs at the least substituted C–H site next to the ketone (black arrow). Our ML model predicts a p*K*_a_ value of 24.7 for the experimental site of reaction. Also, our ML model predicts that the reaction site should be at the highlighted circle. For this site, the ML model predicts a p*K*_a_ value of 16.4. It is generally accepted that the most substituted C–H site next to a ketone will form the more stable carbanion (thermodynamic anion), whereas the least substituted carbanion will be the least stable carbanion (kinetic anion). This can generally be controlled by the type of base used. For the reaction in Scheme 1a, *n*-BuLi is commly used, which is known to lead to the kinetic anion. Because our ML model relies on the principle of lowest energy, it predicts the site with the lowest p*K*_a_ value as the site of reaction (thermodynamic carbanion) and does not account for the type of base used.

Going to Scheme 1b, we look at a Claisen reaction where the experimental site of reaction occurs at the least substituted ketone. Our ML model predicts the p*K*_a_ value here to be 20.5; however, the lowest ML-predicted p*K*_a_ value is 4.2. Again, the ML model correctly predicts the most stable carbanion (lowest p*K*_a_ value), but other factors come into play when synthesizing compounds.

Last, we have an example of the Michael reaction in Scheme 1c. Here, both the experimental site of reaction and the ML-predicted site of reaction match. Our ML model predicts the lowest p*K*_a_ value to be 12.5, whereas the second lowest ML-predicted p*K*_a_ value is 21.9 (the least substituted C–H next to a ketone). For more information, see Supporting Information File 1, section “Outliers for Reaxys”.

When we evaluate our ML models on the whole out-of-sample set, we again find that the regression model (MCC of 0.82) outperforms the classification model (MCC of 0.70) when used as a binary classifier as seen in Table 2. While a direct comparison cannot be made between Roszal et al.’s results [3] and ours, we find our result to outperform theirs with an accuracy of 0.96. In general, it is surprising that the LightGBM regressor outperforms our LightGBM classifier as Ree et al. [14] have shown the opposite to be true for electrophilic aromatic substitutions. However, our regression model serves a dual function, that is, it accurately predicts p*K*_a_ values and identifies the reaction site.

### Prediction of aryl C–H borylation sites

**Figure 3:**
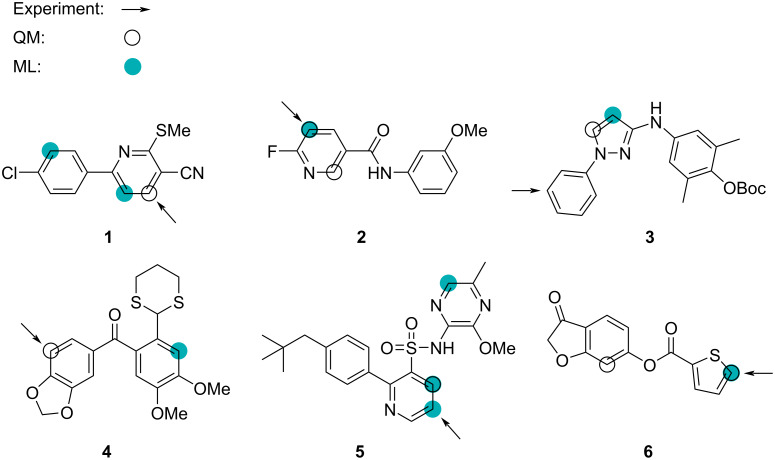
Predicting the site of borylation for a set of six experimentally reported borylation reactions [45]. Arrow: major experimental site/prediction by SoBo; black ring: QM-computed lowest p*K*_a_ + 1.5; teal filled circle: ML-predicted lowest p*K*_a_ + 1.5.

In the previous section, we showed that our ML model is able to predict the reaction site for p*K*_a_-dependent reactions. Now, we test the ML model on a more complex reaction type, namely, borylation reactions. Caldeweyher et al. [45] presented a workflow to predict the iridium-catalyzed borylation site of aryl C–H bonds (SoBo) [45] and experimentally validated their approach using six pharmaceutical intermediates from medicinal chemistry programs. In the article, they state that *”Iridium catalysts ligated by bipyridine ligands catalyze the borylation of the aryl C–H bonds that are most acidic and least sterically hindered…”*[45]. For this reason, we tested both our QM workflow and the ML model to see how well they identify the reaction site when only considering the lowest aromatic C–H p*K*_a_ value; see Figure 3. For both methods, we identify the possible site of reaction if the p*K*_a_ value is within 1.5 p*K*_a_ units of the lowest p*K*_a_ value. This is slightly different from our previous approach. However, because of the higher complexity of the reaction and the similarity of aromatic C–H sites, we purposely allow the QM workflow and the ML model to assess more sites as ‘1’ or true site. When the p*K*_a_ value is within 1.5 p*K*_a_ units, we also ensure that we are within the range or the uncertainty of the QM-computed p*K*_a_ values, which have a MAE of 1.48, as discussed in section “Computing p*K*_a_ values”.

For compound **1**, the ML model predicts two low-p*K*_a_ sites, indicated by filled circles, of which none corresponds to the experimentally observed site of borylation, indicated by the arrow. However, the QM workflow predicts the correct site as the black ring indicates. Overall, the QM workflow accurately predicts four of the six borylation sites, although, in the case of compounds **2** and **6**, there are additional sites with nearly identical p*K*_a_ values. In the case of compound **3**, most chemists would expect the p*K*_a_ of pyrazole C–H sites to be considerably lower than those on the benzene ring, suggesting that factors other than p*K*_a_ determine the site of borylation for this compound. In the case of compound **5**, the most likely explanation is that the site with the lowest QM-computed p*K*_a_ value is sterically hindered compared to the experimentally observed site of borylation. The ML model predicts three borylation sites correctly, but, in the case of compound **5**, there are two additional sites with low p*K*_a_ values. One failure is for compound **3**, where the QM workflow also fails; however, for compounds **1** and **4**, the ML model fails, while the QM workflow accurately predicts the site of borylation. This indicates that these compounds are not well represented in the training set.

## Conclusion

We introduce pKalculator, an automated QM-based workflow that computes C–H p*K*_a_ values with a MAE of 1.48 and a RMSE of 1.81 when correlating with experimental p*K*_a_ values. We use this method to generate training data for an atom-based regression model that delivers fast and relatively precise predictions with MAE and RMSE values of 1.24 and 2.15, respectively, when correlating with QM-computed p*K*_a_ values. Both methods are freely available under the MIT license. Our workflow can function as a filtering tool for computer-aided synthesis planning for the synthesis of various p*K*_a_-dependent reactions (aldol, Michael, and Claisen), evidenced by its accurate predictions of reaction sites for 1043 reactions (MCC of 0.82). Looking ahead, we aim to explore more reactions that depend on C–H p*K*_a_ values, further enhancing the utility of pKalculator for synthetic chemists. Future iterations will consider factors such as a more extensive and diverse training set, as well as steric hindrance and base reactivity, ensuring even more precise predictions for reaction sites.

## Supporting Information

File 1 Additional methods data.

## Data Availability

All data that supports the findings of this study is available in the published article and/or the supporting information to this article. The code for the automated workflow and results of the analyzed data are available at https://github.com/jensengroup/pKalculator. Aditional data is available at https://sid.erda.dk/sharelink/EyuyjllJdp. The internet Bond-energy Databank (iBonD) is accessible for non-profit academic use. Due to licensing restrictions for Reaxys, the Reaxys data cannot be shared. We have provided a list of reaction IDs together with our predictions.
