# Supplementary material for: pKalculator: A pKa predictor for C–H bonds
Source: Beilstein J Org Chem. 2024 Jul 16;20:1614–22. doi: 10.3762/bjoc.20.144 (PMC11285060; doi:10.3762/bjoc.20.144)
Supplement: File 1 — Additional methods data. [file Beilstein_J_Org_Chem-20-1614-s001.pdf]

BEILSTEIN JOURNAL OF ORGANIC CHEMISTRY

Supporting Information

for

pKalculator: A pK_a predictor for C–H bonds

Rasmus M. Borup, Nicolai Ree and Jan H. Jensen

Beilstein J. Org. Chem. **2024,** *20,* 1614–1622. doi:10.3762/bjoc.20.144

Additional methods data

License and Terms: This is a supporting information file under the terms of the Creative Commons Attribution License (https://creativecommons.org/licenses/by/4.0). Please note that the reuse, redistribution and reproduction in particular requires that the author(s) and source are credited and that individual graphics may be subject to special legal provisions.
The license is subject to the *Beilstein Journal of Organic Chemistry* terms and conditions: (https://www.beilstein-journals.org/bjoc/terms)

Selecting unique conformers

Conformers with relative energies above 3 kcal/mol are removed from the conformer selection when finding the unique conformers as they are considered too high in energy compared to the rest. Hereafter, the similarity of conformers are compared using a distance (RMS) matrix of the conformers of a molecule. Now, the unsupervised non-hierarchical Butina clustering algorithm is used. Each cluster centroid is the conformer with the largest number of neighbors, and the neighbors have a distance threshold of 0.5 Å. Hereafter, the conformers are sorted by the number of neighbors, and the first conformer in the list (centroid) is selected. Each conformer inside the distance threshold of 0.5 Å becomes part of the same cluster and is removed from the list. This is repeated for the rest of the list, and conformers that are not part of a cluster become single instances [1].

Benchmark study - computational methods

This benchmark study evaluates the computational effort and the accuracy of single-point calculations or re-optimizations based on `GFN2-xTB`. These calculations utilize ORCA (v. 5.0.4) [2,3] with DMSO as the solvent. The composite electronic structure method $\mathtt{r^2SCAN\text{-}3c}$ [4], combined with its custom def2-mTZVPP/J basis set and the universal solvation model (SMD) [5], is employed.

Furthermore, calculations using density functional theory (DFT) are conducted. For this purpose, the dispersion D4-corrected DFT functional CAM-B3LYP [6,7], the Karlsruhe triple-ζ basis set (def2-TZVPPD) [8,9], and the conductor-like polarizable continuum model (CPCM) [10] implicit solvation model are employed. An unpublished previous benchmark study indicates that a long-range-corrected functional with dispersion correction excels in computing anions. This trend is also observed here, with CAM-B3LYP D4 showing the best performance, as demonstrated in Figure S1, Table S1, and Table S2. Figures S2–S6 show different linear regressions between $\Delta G^{\circ}_{\mathrm{min}}$ values at T = 295.15 K against experimental pK_{a} for the Bordwell dataset (419 compounds) for the different QM methods.

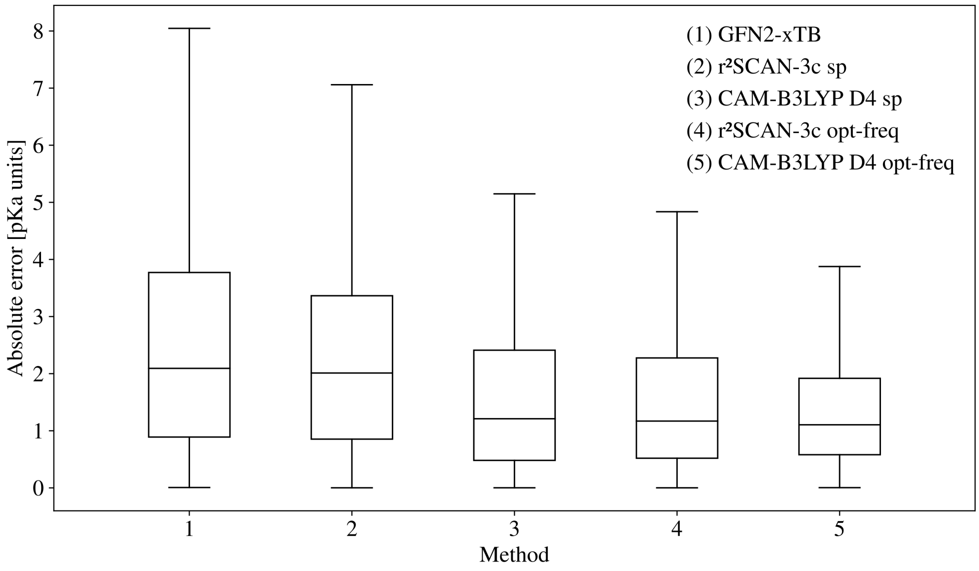

Figure S1: Absolute pK_a unit errors for different levels of theory using the Bordwell dataset (419 compounds). (1) GFN2-xTB ALPB(DMSO); (2) r^2SCAN-3c SMD(DMSO)/GFN2-xTB ALPB(DMSO); (3) CAM-B3LYP/def2-TZVPPD CPCM(DMSO)/GFN2-xTB ALPB(DMSO); (4) r^2SCAN-3c SMD(DMSO))//GFN2-xTB ALPB(DMSO); (5) CAM-B3LYP/def2-TZVPPD CPCM(DMSO)//GFN2-xTB ALPB(DMSO).

Table S1: Absolute pK_a unit error metrics for different levels of theory using the Bordwell dataset (419 compounds). (1) GFN2-xTB ALPB(DMSO); (2) r^2SCAN-3c SMD(DMSO)/GFN2-xTB ALPB(DMSO); (3) CAM-B3LYP/def2-TZVPPD CPCM(DMSO)/GFN2-xTB ALPB(DMSO); (4) r^2SCAN-3c SMD(DMSO))//GFN2-xTB ALPB(DMSO); (5) CAM-B3LYP/def2-TZVPPD CPCM(DMSO)//GFN2-xTB ALPB(DMSO).

	(1)	(2)	(3)	(4)	(5)
mean	2.66	2.35	1.72	1.63	1.51
std	2.27	1.90	1.73	1.64	1.49
min	$6.30 \cdot 10^{-3}$	$1.40 \cdot 10^{-3}$	$1.80 \cdot 10^{-3}$	$9.00 \cdot 10^{-4}$	$4.10 \cdot 10^{-3}$
25%	0.89	0.85	0.48	0.52	0.58
50%	2.09	2.01	1.21	1.17	1.10
75%	3.77	3.36	2.41	2.28	1.92
max	13.46	11.68	10.72	10.36	10.68

Table S2: Summary of the benchmark study for the Bordwell dataset (419 compounds). r^2: coefficient of determination; ρ: Spearman's rank correlation coefficient; MAE: mean absolute error; RMSE: root mean squared error; reg: linear regression.

Functional	Basis set	sp / opt-freq	MAE	RMSE	r^2	ρ	reg
GFN2-xTB	-	-	2.66	3.49	0.81	0.91	$0.41x - 40.25$
r^2SCAN-3c	def2-mTZVPP/J	single-point	2.36	3.02	0.86	0.92	$0.59x - 164.31$
CAM-B3LYP D4	def2-TZVPPD	single-point	1.71	2.43	0.91	0.95	$0.63x - 176.99$
r^2SCAN-3c	def2-mTZVPP/J	opt-freq	1.63	2.31	0.92	0.96	$0.60x - 160.92$
CAM-B3LYP D4	def2-TZVPPD	opt-freq	1.51	2.12	0.93	0.96	$0.62x - 165.58$

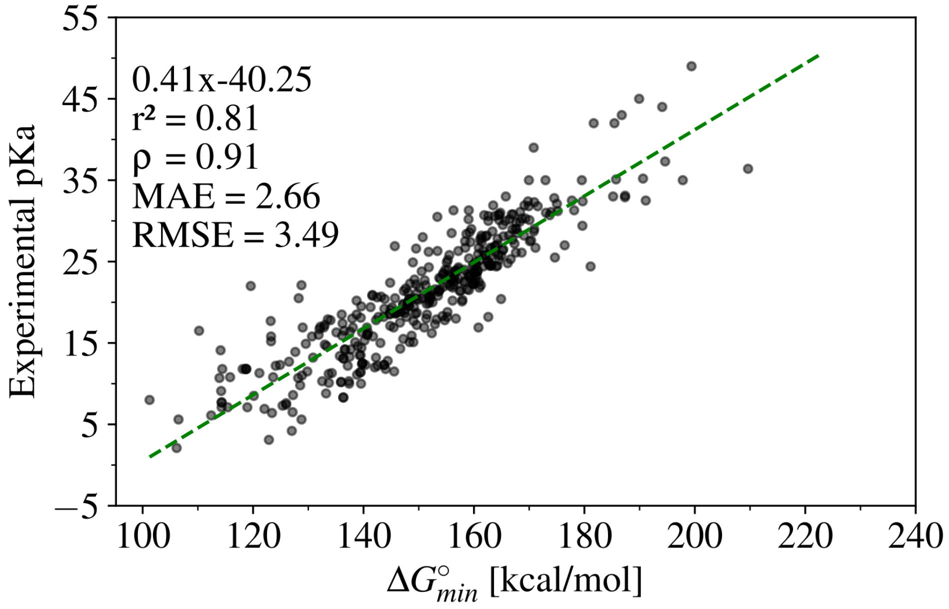

Figure S2: $\Delta G^{\circ}_{\text{min}}$ values at T = 295.15 K against experimental pK_{a} for the Bordwell dataset (419 compounds). pK_{a} = $0.41 \cdot \Delta G^{\circ} - 40.25$; r^2: coefficient of determination; ρ: Spearman's rank correlation coefficient; MAE: mean absolute error; RMSE: root mean squared error. All calculations are carried out at the GFN2-xTB ALPB(DMSO) level of theory.

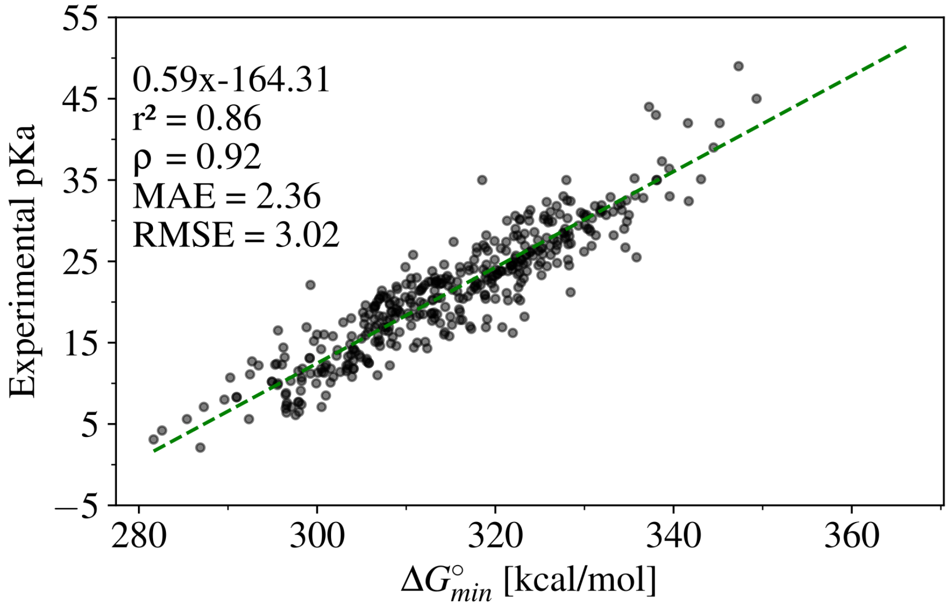

Figure S3: $\Delta G^{\circ}_{\text{min}}$ values at T = 295.15 K against experimental pK_{a} for the Bordwell dataset (419 compounds). pK_{a} = $0.59 \cdot \Delta G^{\circ} - 164.31$; r^2: coefficient of determination; ρ: Spearman's rank correlation coefficient; MAE: mean absolute error; RMSE: root mean squared error. All calculations are carried out at the r^2SCAN-3c SMD(DMSO))/GFN2-xTB ALPB(DMSO) level of theory.

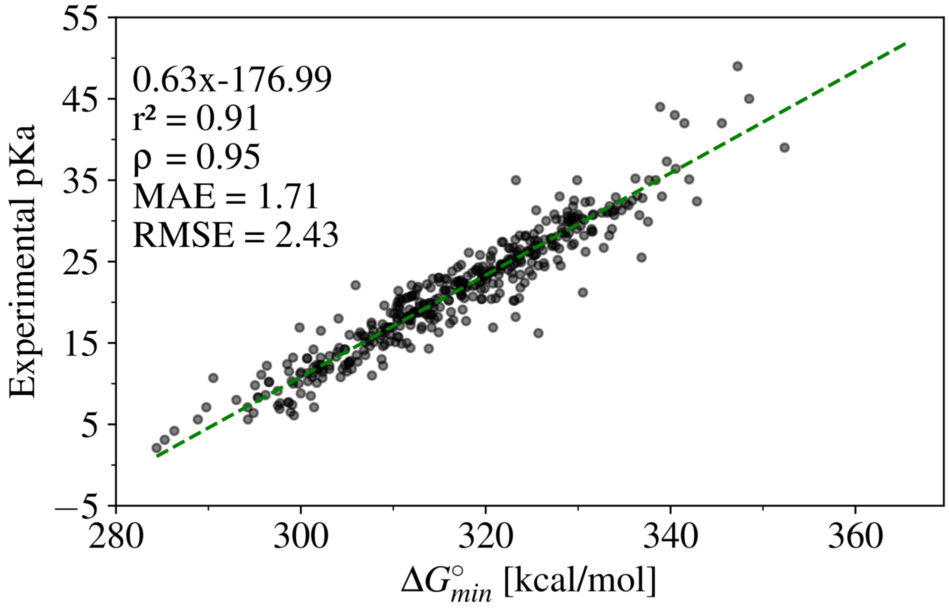

Figure S4: $\Delta G^{\circ}_{\mathrm{min}}$ values at T = 295.15 K against experimental pK_{a} for the Bordwell dataset (419 compounds). pK_{a} = 0.63 · ΔG° − 176.99; r^2: coefficient of determination; ρ: Spearman's rank correlation coefficient; MAE: mean absolute error; RMSE: root mean squared error. All calculations are carried out at the CAM-B3LYP/def2-TZVPPD CPCM(DMSO)/GFN2-xTB ALPB(DMSO) level of theory.

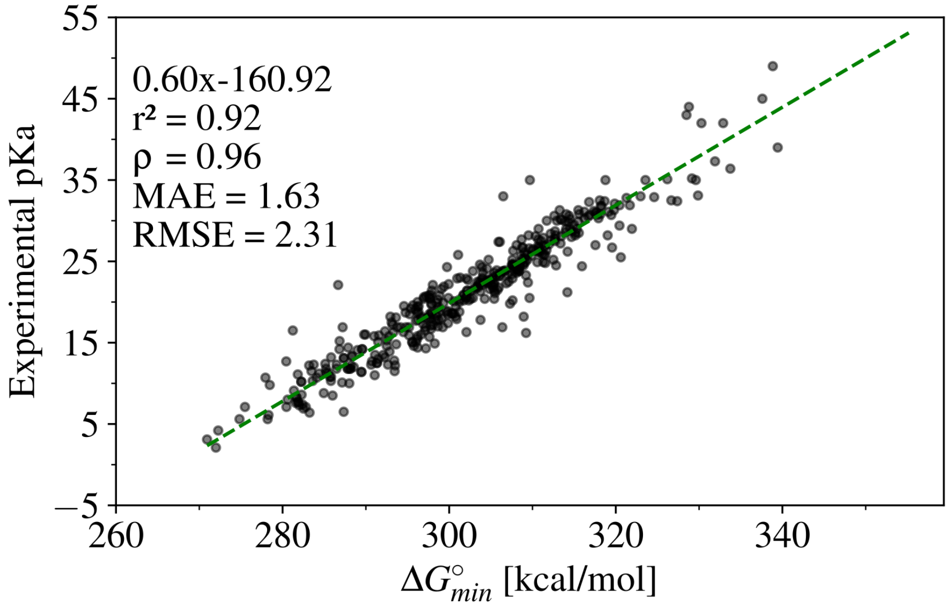

Figure S5: $\Delta G^{\circ}_{\mathrm{min}}$ values at T = 295.15 K against experimental pK_{a} for the Bordwell dataset (419 compounds). pK_{a} = 0.60 · ΔG° − 160.92; r^2: coefficient of determination; ρ: Spearman's rank correlation coefficient; MAE: mean absolute error; RMSE: root mean squared error. All calculations are carried out at the r^2SCAN-3c SMD(DMSO))//GFN2-xTB ALPB(DMSO) level of theory.

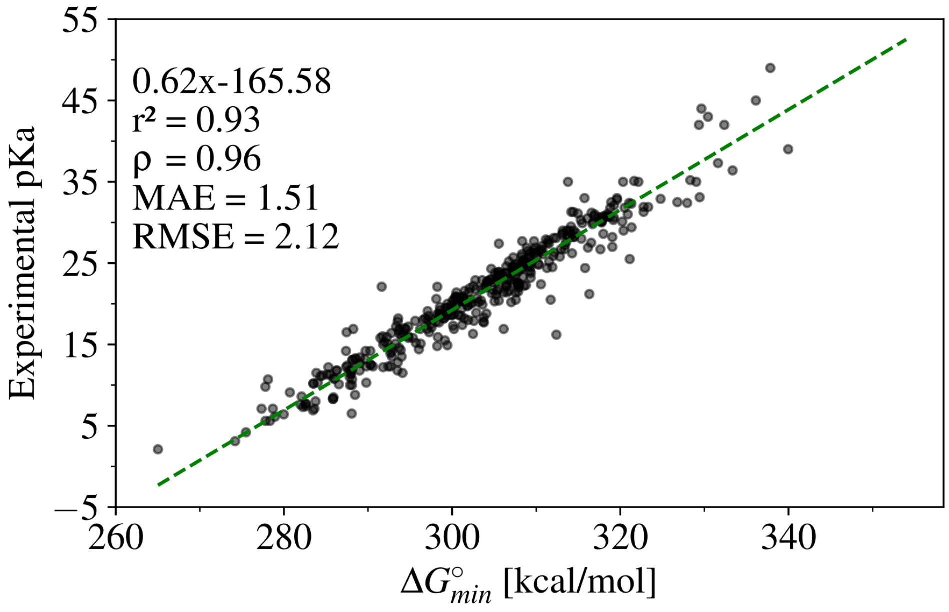

Figure S6: $\Delta G^{\circ}_{\mathrm{min}}$ values at T = 295.15 K against experimental pK_{a} for the Bordwell dataset (419 compounds). pK_{a} = $0.62 \cdot \Delta G^{\circ} - 165.58$; r^2: coefficient of determination; ρ: Spearman's rank correlation coefficient; MAE: mean absolute error; RMSE: root mean squared error. All calculations are carried out at the CAM-B3LYP/def2-TZVPPD CPCM(DMSO)//GFN2-xTB ALPB(DMSO) level of theory.

Finding outliers

Of the 775 molecules, 43 compounds are from [11] with no experimental pK_a values. 732 compounds are therefore left to find the linear relationship between the experimental pK_a values and the lowest ΔG° values, see Figure S7.

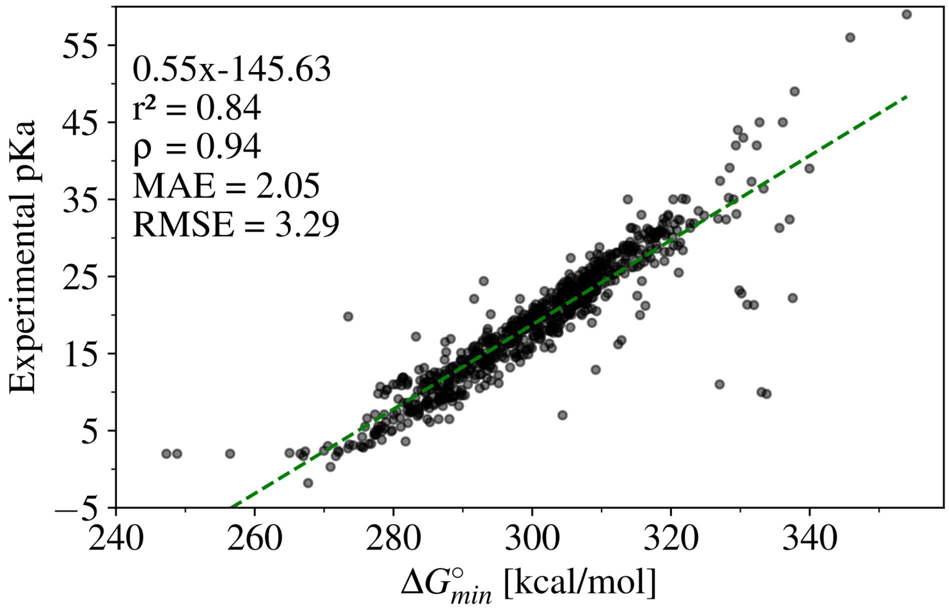

Figure S7: $\Delta G^\circ_{\mathrm{min}}$ values at T = 295.15 K against experimental pK_a for 735 compounds. pK_a = $0.55 \cdot \Delta G^\circ - 145.63$; r^2: coefficient of determination; ρ: Spearman's rank correlation coefficient; MAE: mean absolute error; RMSE: root mean squared error. All calculations are carried out at the CAM-B3LYP/def2-TZVPPD CPCM(DMSO)//GFN2-xTB ALPB(DMSO) level of theory.

Outliers with an error exceeding 5 pK_a units are reviewed for calculation errors or errors in the literature. The observed outliers typically result from one of the following reasons: calculation errors concerning the expected minimum pK_a site, discrepancies between literature structures and database structures, mislabeled experimental pK_a values, or extrapolated pK_a values. Notably, the extrapolated pK_a values correspond to compounds beyond the scale (pK_a $\geq$ 35) measurable in DMSO because of DMSO's autoprotolysis (pK_a = 35) [12,13]. The final result consists of 695 molecules, which can be seen in Figure S8 where outliers have been omitted. Table S3 shows the different compound names omitted from the linear regression in Figure S8. It should also be noted that we altered our QM workflow for compounds that exhibit bridgeheads. Instead of

generating $\min(1 + 3n_{\text{rot}}, 20)$ conformers for each SMILES using RDKit (v.2022.09.4) [14], we generate $\min(10 + 3n_{\text{rot}}, 20)$ conformers for the neutral SMILES. Hereafter, we deprotonate each C–H bond for each generated neutral conformer and pass it through the QM workflow. The reason for doing this is that our original QM workflow sometimes shortlists deprotonated conformers that are very different from the neutral conformers. This produces high energy differences that yield erroneous pK_a values.

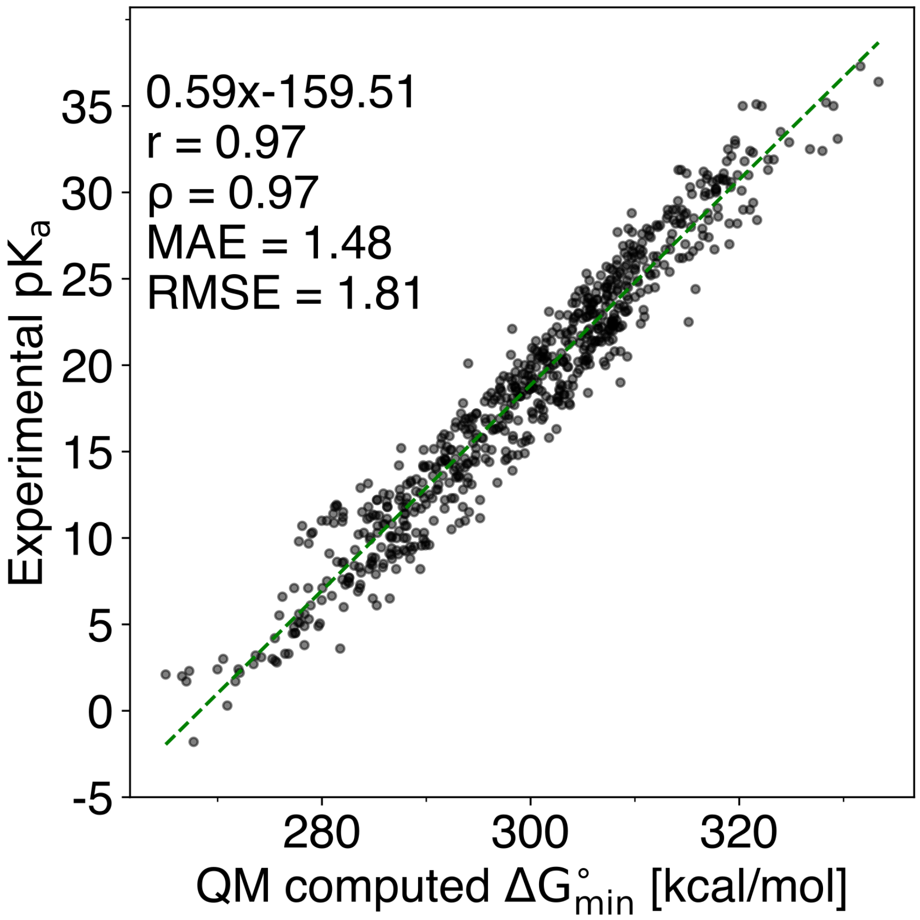

Figure S8: $\Delta G^{\circ}_{\text{min}}$ values at $T = 295.15$ K against experimental pK_a for 695 compounds. p$K_a = 0.59 \cdot \Delta G^{\circ} - 159.51$; r: Pearson correlation coefficient; ρ: Spearman's rank correlation coefficient; MAE: mean absolute error; RMSE: root mean squared error. All calculations are carried out at the CAM-B3LYP/def2-TZVPPD CPCM(DMSO)//GFN2-xTB ALPB(DMSO) level of theory.

Table S3: Outliers for the Bordwell and iBond datasets (n = 37). Estimated : extrapolated pk_a values (n = 21); high error: high errors on the expected lowest pK_a site compared to the calculated values (n = 12); wrong site: failed computation on the expected lowest pK_a site (n = 4).

names	outlier note
comp301	estimated
ibond25	estimated
ibond163	estimated
ibond204	estimated
comp419	estimated
ibond211	estimated
comp390	estimated
ibond216	estimated
ibond233	estimated
comp315	estimated
ibond17	estimated
comp300	estimated
comp290	estimated
comp217	estimated
comp195	estimated
comp194	estimated
comp193	estimated
ibond240	estimated
ibond241	estimated
ibond244	estimated
ibond20	estimated
comp57	high error
ibond24	high error

ibond347	high error
ibond235	high error
ibond175	high error
ibond10	high error
comp405	high error
comp338	high error
comp325	high error
comp339	high error
comp158	high error
comp103	high error
ibond323	wrong site
ibond194	wrong site
ibond4	wrong site
ibond330	wrong site

The descriptor

The following section evaluates which descriptor vector best describes our pK_a values. From Table S4 and Figure S9, it is seen that the charge shell descriptor with six shells and values sorted according to the Cahn—Ingold—Prelog (CIP) rules best describes the pK_a values.

Table S4: Performance metrics using different numbers of shells (n-shells) for the descriptor vector for the Bordwell dataset (419 compounds). MAE: mean absolute error; RMSE: root mean squared error.

Descriptor	n-shells	Dimensions	MAE	RMSE
Sorted-shell	3	53	2.03	3.39
Sorted-shell	4	161	1.98	3.32
Sorted-shell	5	485	1.90	3.08
**Sorted-shell**	**6**	**1457**	**1.82**	**2.95**
Sorted-shell	7	4373	1.82	2.95

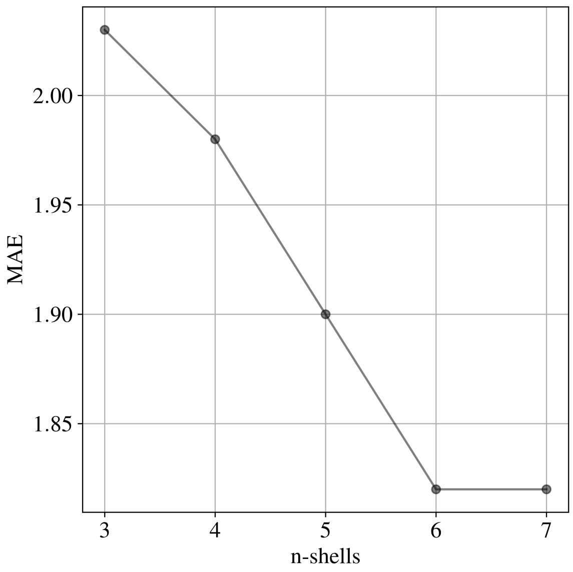

(a) MAE vs n-shells

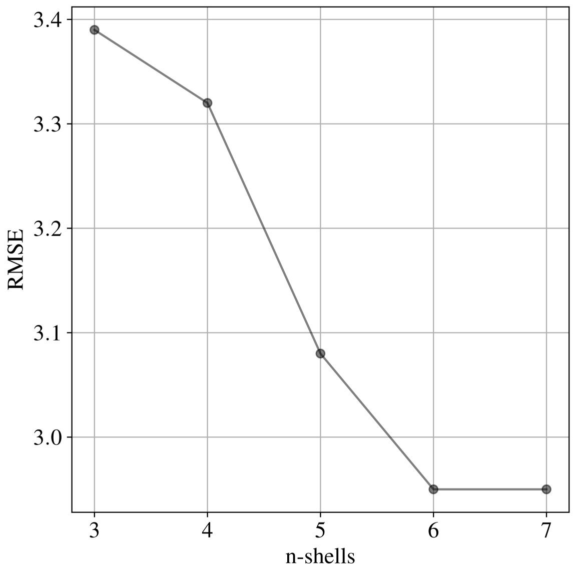

(b) RMSE vs n-shells

Figure S9: MAE versus n-shells and RMSE versus n-shell for the Bordwell dataset (419 compounds) MAE: mean absolute error; RMSE: root mean squared error.

Machine learning models

Regression and binary classification models have been trained to evaluate either how well we can predict pK_a values for each site or how well we can predict the site of reaction. Table S5 shows the performance metrics for the different regression models using fivefold cross-validation.

Based on the classification of the minimum pK_a value for each site in a molecule is set to either a ‘1’ (lowest pK_a site) or ‘0’ (not lowest pK_a site; we also introduce a tolerance where a pK_a value within +1 pK_a units or +2 pK_a units of the lowest pK_a value is accepted as ‘1’ to account for slight variations; see Table S6. From that, a confusion matrix (CM) is constructed to compare the predictions of the machine learning (ML) model to the calculated sites. A site is classified as a true positive (TP) or true negative (TN) if the ML model’s prediction aligns with the QM-computed sites. Conversely, it is labeled as a false positive (FP) or false negative (FN) if the ML prediction differs from the calculated sites. From these classifications, we derive several evaluation parameters: accuracy (ACC), Matthew’s correlation coefficient (MCC), recall/sensitivity (true-positive rate -

TPR), specificity (true-negative rate - TNR), precision (positive predictive value - PPV), and negative predictive value (NPV).

Table S5: Performance metrics for different regression models. The dataset (775 compounds; 3910 pK_a values) is randomly split into a training set (80%; 620 compounds; 3121 pK_a values) and a held-out test set (20%; 155 compounds; 789 pK_a values). Subsequently, a fivefold randomly shuffled cross-validation (CV) is conducted. Within each fold, the original training set is split randomly into a new training set (90% of the original training set) and a validation set (10% of the original training set). Hereafter, each ML model is trained on our original training set (80%; 620 compounds; 3121 pK_a values) and tested against the held-out test set (20%; 155 compounds; 789 pK_a values). μ MAE: mean mean absolute error for fivefold CV; μ RMSE: mean root mean squared error for fivefold CV; MAE: mean absolute error; RMSE: root mean squared error for fivefold CV.

Method	Train		Valid		Test	
Regression	μ MAE	μ RMSE	μ MAE	μ RMSE	MAE	RMSE
LightGBM GBDT (default)	0.16 ± 0.08	0.25 ± 0.11	1.24 ± 0.14	2.18 ± 0.31	1.37	2.45
LightGBM GBDT	0.37 ± 0.06	0.57 ± 0.09	1.25 ± 0.12	2.2 ± 0.30	1.31	2.20
LightGBM DART (default)	$0.06 \pm 1.7 \cdot 10^{-3}$	$0.10 \pm 7.3 \cdot 10^{-3}$	1.13 ± 0.12	2.04 ± 0.30	1.24	2.24
**LightGBM DART**	$0.08 \pm 1.4 \cdot 10^{-3}$	$0.14 \pm 5.3 \cdot 10^{-3}$	1.13 ± 0.11	2.01 ± 0.32	**1.24**	**2.15**

Table S6: Examples for targets in binary classification. '1' is given for the lowest pK_a value, and '0' is given for all other calculated pK_a values. +1 and +2 denotes either +1 pK_a units or +2 pK_a units of the lowest pK_a value accepted as '1' (true site).

pK_a values	target	taget+1	taget+2
[15, 20, 25]	[1, 0, 0]	[1, 0, 0]	[1, 0, 0]
[10, 5, 3]	[0, 0, 1]	[0, 0, 1]	[0, 1, 1]
[2, 1, 5]	[0, 1, 0]	[1, 1, 0]	[1, 1, 0]

$$\mathrm{ACC} = \frac{\mathrm{TP} + \mathrm{TN}}{\mathrm{TP} + \mathrm{FP} + \mathrm{TN} + \mathrm{FN}} \tag{S1}$$

$$\mathrm{MCC} = \frac{\mathrm{TP} \cdot \mathrm{TN} - \mathrm{FN} \cdot \mathrm{FP}}{\sqrt{(\mathrm{TP} + \mathrm{FN})\,(\mathrm{TP} + \mathrm{FP})\,(\mathrm{FN} + \mathrm{FP})\,(\mathrm{TN} + \mathrm{FN})}} \tag{S2}$$

$$\mathrm{MCC} = \sqrt{\mathrm{PPV} \cdot \mathrm{TPR} \cdot \mathrm{TNR} \cdot \mathrm{NPV}} - \sqrt{(1 - \mathrm{PPV}) \cdot (1 - \mathrm{TPR}) \cdot (1 - \mathrm{TNR}) \cdot (1 - \mathrm{NPV})} \tag{S3}$$

$$\text{Sensitivity} = \text{Recall} = \text{TPR} = \frac{\text{TP}}{\text{TP} + \text{FN}} \tag{S4}$$

$$\text{Specificity} = \text{TNR} = \frac{\text{TN}}{\text{TN} + \text{FP}} \tag{S5}$$

$$\text{Precision} = \text{PPV} = \frac{\text{TP}}{\text{TP} + \text{FP}} \tag{S6}$$

$$\text{NPV} = \frac{\text{TN}}{\text{TN} + \text{FN}} \tag{S7}$$

Table S7: Training and validation set performance metrics for different binary classification models. The dataset (775 compounds; 3910 pK_a values) is randomly split into a training set (80%; 620 compounds; 3121 pK_a values) and a held-out test set (20%; 155 compounds; 789 pK_a values). Subsequently, a fivefold randomly shuffled cross-validation (CV) is conducted. Within each fold, the original training set is split randomly into a new training set (90% of the original training set) and a validation set (10% of the original training set). +1 and +2 denotes either +1 pK_a units or +2 pK_a units of the lowest pK_a value accepted as '1' (true site). AUC: Area under the curve.

Method	training set		validation set	
Classification	mean AUC	mean logloss	mean AUC	mean logloss
LightGBM GBDT (default)	1.00	0.01	0.99	0.10
LightGBM DART (default)	1.00	0.00	0.99	0.29
LightGBM GBDT	1.00	0.01	0.99	0.10
LightGBM DART	1.00	0.01	0.99	0.10
	-	-	-	-
LightGBM GBDT +1 (default)	1.00	0.01	0.99	0.10
LightGBM DART +1 (default)	1.00	0.00	0.98	0.29
LightGBM GBDT +1	1.00	0.04	0.99	0.10
LightGBM DART +1	1.00	0.02	0.99	0.09
	-	-	-	-
LightGBM GBDT +2 (default)	1.00	0.01	0.99	0.11
LightGBM DART +2 (default)	1.00	0.00	0.98	0.31
LightGBM GBDT +2	1.00	0.02	0.99	0.10
LightGBM DART +2	1.00	0.02	0.99	0.12

Table S8: Held-out test set performance metrics for different binary classification models, including the best regression model. The dataset (775 compounds; 3910 pK_a values) is randomly split into a training set (80%; 620 compounds; 3121 pK_a values) and a held-out test set (20%; 155 compounds; 789 pK_a values). Each pK_a value in a molecule is set to either a '1' (lowest pK_a site) or '0' (not lowest pK_a site. +1 and +2 denotes either +1 pK_a units or +2 pK_a units of the lowest pK_a value accepted as '1' (true site). For the null models, all sites are set to '0'. The best models are marked in **bold**.

Method	ACC	MCC	PPV	TPR	TNR	NPV
Null model	0.80	0.00	0.00	0.00	1.00	0.80
LightGBM GBDT (default)	0.97	0.91	0.97	0.90	0.99	0.98
LightGBM DART (default)	0.96	0.88	0.93	0.88	0.98	0.97
LightGBM GBDT	0.97	0.92	0.97	0.90	0.99	0.98
LightGBM DART	0.96	0.88	0.90	0.90	0.98	0.97
**LightGBM DART reg**	**0.99**	**0.97**	**0.97**	**0.97**	**0.99**	**0.99**
	-	-	-	-	-	-
Null model +1	0.80	0.00	0.00	0.00	1.00	0.80
LightGBM GBDT (default) +1	0.97	0.90	0.94	0.90	0.99	0.97
LightGBM DART (default) +1	0.96	0.86	0.90	0.88	0.97	0.97
LightGBM GBDT +1	0.96	0.86	0.89	0.89	0.97	0.97
LightGBM DART +1	0.96	0.88	0.90	0.91	0.97	0.98
**LightGBM DART +1 reg**	**0.99**	**0.97**	**0.97**	**0.98**	**0.99**	**1.00**
	-	-	-	-	-	-
Null model +2	0.80	0.00	0.00	0.00	1.00	0.80
LightGBM GBDT (default) +2	0.96	0.86	0.89	0.89	0.97	0.97
LightGBM DART (default) +2	0.96	0.86	0.89	0.89	0.97	0.97
LightGBM GBDT +2	0.96	0.86	0.89	0.89	0.97	0.97
LightGBM DART +2	0.96	0.86	0.89	0.89	0.97	0.97
**LightGBM DART +2 reg**	**0.98**	**0.95**	**0.95**	**0.97**	**0.99**	**0.99**

Table S9: Held-out test set performance metrics for different binary classification models, including the best regression model. The dataset (775 compounds; 3910 pK_a values) is randomly split into a training set (80%; 620 compounds; 3121 pK_a values) and a held-out test set (20%; 155 compounds; 789 pK_a values). Each pK_a value in a molecule is set to either a '1' (lowest pK_a site) or '0' (not lowest pK_a site. +1 and +2 denotes either +1 pK_a units or +2 pK_a units of the lowest pK_a value accepted as '1' (true site). For the null models, all sites are set to '0'. The best models are marked in **bold**.

Method	TP	TN	FP	FN
Null model	0	634	0	155
LightGBM GBDT (default)	139	629	5	16
LightGBM DART (default)	136	623	11	19
LightGBM GBDT	139	630	4	16
LightGBM DART	139	619	15	16
**LightGBM DART reg**	**151**	**630**	**4**	**4**
	-	-	-	-
Null model +1	0	632	0	157
LightGBM GBDT (default) +1	141	623	9	16
LightGBM DART (default) +1	138	616	16	19
LightGBM GBDT +1	140	615	17	17
LightGBM DART +1	143	616	16	14
**LightGBM DART +1 reg**	**154**	**628**	**4**	**3**
	-	-	-	-
Null model +2	0	629	0	160
LightGBM GBDT (default) +2	138	618	11	22
LightGBM DART (default) +2	139	612	17	21
LightGBM GBDT +2	142	615	14	18
LightGBM DART +2	144	609	20	16
**LightGBM DART +2 reg**	**155**	**620**	**9**	**5**

Outliers for the test set

This section examines the outliers associated with the test set for the best regression model; see Table S10. Generally, the regression model struggles to accurately predict the C–H pK_a values at bridgehead positions (comp69, comp70, comp321). Bridgehead deprotonation is often energetically unfavorable, leading to an unstable anion. Since our descriptor vector solely describes CM5 charges, it fails to account for the steric strain associated with the bridgehead position. The regression model also encounters difficulties when an anion gains extra stability through charge delocalization via resonance, as shown in Figure S10. It is expected that our model may struggle to describe the additional stability arising from conjugation since the descriptor vector for CM5 charges originates from the neutral molecule. A more accurate approach to generate the descriptor vector could involve concatenating the CM5 charges for each deprotonated site within the molecule or by taking the difference between the neutral descriptor vector and the deprotonated descriptor vector for each site, thereby providing a more precise representation of the molecule.

Table S10: Outliers (n = 15) from the held-out test set (20%; 155 compounds; 789 pK_a values) using the best regression model. The outliers below have an error of seven or above between the calculated (QM) and predicted (ML) sites. * denotes the lowest pK_a site, and ** denotes bridgehead sites.

names	atom site	pk$_a$ exp	pk$_a$ calc	pk$_a$ pred	error pred vs calc
comp69	7**	29.0	43.7	30.5	13.2
comp70	7**	28.1	46.5	30.3	16.2
comp89	3*	10.7	5.8	14.5	8.7
comp215	1*	13.2	12.7	23.0	10.3
comp226	3*	18.0	16.8	31.2	14.4
comp227	9*	26.1	25.3	32.6	7.3
comp284	1	24.0	33.8	26.6	7.1
comp284	4	24.0	33.8	26.3	7.5
comp284	9	24.0	23.5	30.9	7.3
comp321	6**	30.5	35.9	44.2	8.3
ibond242	13*	18.2	18.1	29.6	11.4
ibond270	5*	12.6	12.6	5.0	7.6
ibond297	2*	28.4	31.8	39.7	8.0
ibond320	6*	29.9	29.1	36.6	7.6
ibond194	5*	31.3	26.7	36.9	10.2

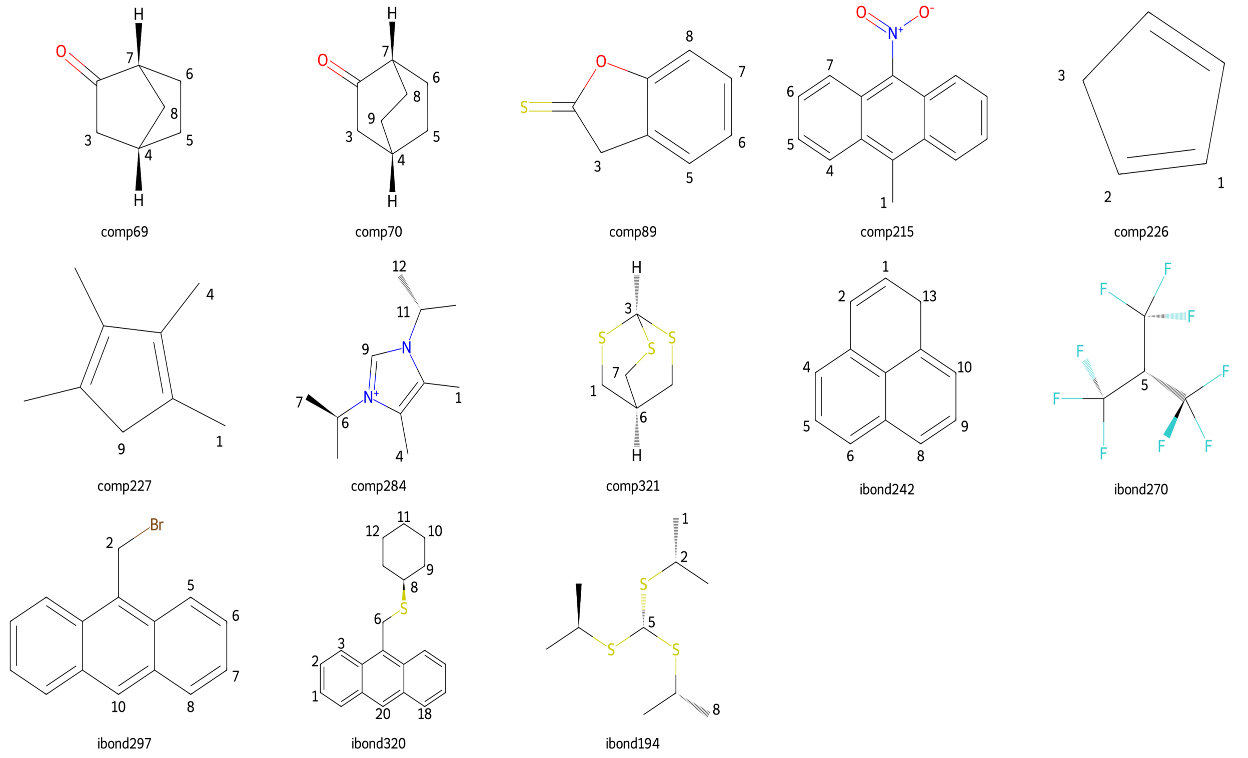

Figure S10: Outliers from the held-out test set (20%; 155 compounds; 789 pK_a values) using the best regression model. The outliers below have an error of seven or above between the calculated (QM) and predicted (ML) sites.

Similarly, when looking at the false positives (FP) and false negatives (FN) from the binary classification of the lowest pK_a site for the held-out test set (20%; 155 compounds; 789 pK_a values), the following sites emerge as troublesome; see Table S11 and Figure S11.

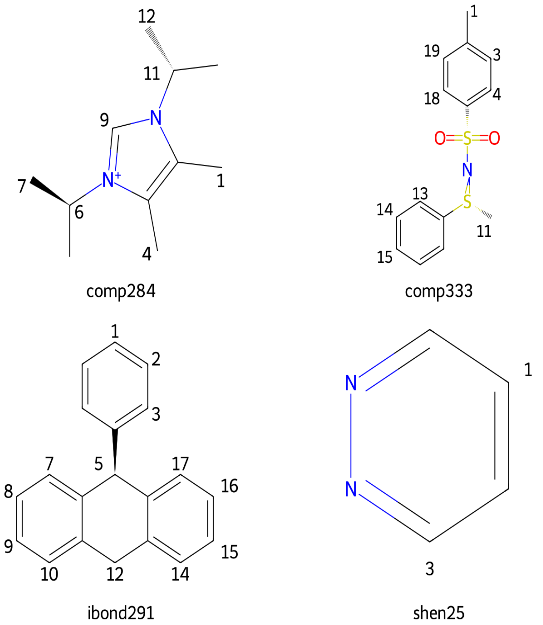

Figure S11: False negative (FN) and false positives (FP) from the held-out test set (20%; 155 compounds; 789 pK_a values) using the best regression model as a binary classifier.

Table S11: False positives (FP, n = 4) and false negatives (FN, n = 4) from the held-out test set (20%; 155 compounds; 789 pK_a values) using the best regression model as a binary classifier.

names	atom site	lowest calc	lowest pred	pK_a calc	pK_a pred	err(pred vs calc)
comp284	1	0	0	33.8	26.6	7.1
	4	0	1 (FP)	33.8	26.3	7.5
	6	0	0	39.2	35.6	3.6
	7	-1	-1	inf	inf	inf
	9	1	0 (FN)	23.5	30.9	7.3
	11	0	0	38.8	34.9	3.9
	12	-1	-1	inf	inf	inf
comp333	1	0	1 (FP)	32.2	29.0	3.2
	3	0	0	42.4	41.0	1.4
	4	-1	-1	inf	inf	inf
	11	1	0 (FN)	26.9	30.5	3.5
	13	0	0	35.8	35.9	0.1
	14	0	0	39.9	40.8	0.8
	15	0	0	40.1	40.9	0.7
	18	0	0	41.3	38.4	2.9
	19	0	0	41.7	41.1	0.6
ibond291	1	0	0	43.1	43.4	0.3
	2	0	0	43.2	43.3	0.1
	3	0	0	43.4	43.5	0.1
	5	0	1 (FP)	29.0	26.8	2.1
	7	0	0	43.2	43.9	0.7
	8	0	0	43.3	43.5	0.2
	9	0	0	43.7	44.0	0.3
	10	0	0	43.2	42.8	0.4
	12	1	0 (FN)	28.9	29.2	0.3
	14	0	0	43.5	42.8	0.7
	15	0	0	43.3	44.0	0.7
	16	0	0	43.4	43.9	0.2
	17	0	0	43.2	43.0	0.7
shen25	1	1	0 (FN)	36.2	38.9	2.7
	3	0	1 (FP)	38.6	38.6	0.2

Reaxys

Table S12: Reaxys performance metrics: comparison for different binary classification models, including the best regression model. The Reaxys dataset comprises 1043 reactions (584 aldol, 51 Michael, and 408 Claisen). Each pK_a value in a molecule is set to either a '1' (lowest pK_a site) or '0' (not lowest pK_a site. +1 and +2 denotes either +1 pK_a units or +2 pK_a units of the lowest pK_a value accepted as '1' (true site). For the null models, all sites are set to '0'. The best models are marked in **bold**.

Method	ACC	MCC	PPV	TPR	TNR	NPV
Null model*	0.87	0	0	0	1.00	0.87
LightGBM GBDT (default)	0.92	0.70	0.65	0.84	0.93	0.97
LightGBM DART (default)	0.87	0.56	0.52	0.76	0.89	0.96
LightGBM GBDT	0.92	0.70	0.64	0.87	0.92	0.98
LightGBM DART	0.92	0.69	0.64	0.85	0.93	0.98
**LightGBM DART reg**	**0.96**	**0.82**	**0.84**	**0.84**	**0.98**	**0.98**
	-	-	-	-	-	-
LightGBM GBDT (default) +1	0.91	0.69	0.63	0.86	0.92	0.98
LightGBM DART (default) +1	0.89	0.62	0.55	0.84	0.90	0.97
LightGBM GBDT +1	0.90	0.63	0.60	0.78	0.92	0.96
LightGBM DART +1	0.91	0.68	0.62	0.85	0.92	0.98
**LightGBM DART+1 reg**	**0.96**	**0.82**	**0.82**	**0.86**	**0.97**	**0.98**
	-	-	-	-	-	-
LightGBM GBDT (default) +2	0.92	0.69	0.64	0.85	0.93	0.98
LightGBM DART (default) +2	0.89	0.62	0.55	0.85	0.89	0.97
LightGBM GBDT +2	0.92	0.70	0.64	0.86	0.92	0.98
LightGBM DART +2	0.91	0.67	0.60	0.86	0.91	0.98
**LightGBM DART +2 reg**	**0.96**	**0.82**	**0.80**	**0.88**	**0.97**	**0.98**

Table S13: Reaxys performance metrics: comparison for different binary classification models, including the best regression model. The Reaxys dataset comprises 1043 reactions (584 aldol, 51 Michael, and 408 Claisen). Each pK_a value in a molecule is set to either a '1' (lowest pK_a site) or '0' (not lowest pK_a site. +1 and +2 denotes either +1 pK_a units or +2 pK_a units of the lowest pK_a value accepted as '1' (true site). For the null models, all sites are set to '0'. The best models are marked in **bold**.

Method	TP	TN	FP	FN
Null model	0	6804	0	1050
LightGBM GBDT (default)	884	6332	472	166
LightGBM DART (default)	797	6069	735	253
LightGBM GBDT	917	6285	519	133
LightGBM DART	887	6301	503	163
**LightGBM DART reg**	**880**	**6638**	**166**	**170**
	-	-	-	-
Null model +1	0	6804	0	1050
LightGBM GBDT (default) +1	899	6280	524	151
LightGBM DART (default) +1	878	6098	706	172
LightGBM GBDT +1	814	6278	526	236
LightGBM DART +1	890	6266	538	160
**LightGBM DART +1 reg**	**906**	**6604**	**200**	**144**
	-	-	-	-
Null model +2	0	6804	0	1050
LightGBM GBDT (default) +2	891	6299	505	159
LightGBM DART (default) +2	887	6074	730	163
LightGBM GBDT +2	904	6289	515	146
LightGBM DART +2	901	6211	593	149
**LightGBM DART +2 reg**	**892**	**6574**	**230**	**123**

Outliers for Reaxys

Figure S12 displays a sample of 20 random compounds from the Reaxys dataset, highlighting the limitations of our model in predicting reaction sites based on pK_a-dependent reactions. Because our method relies on the principle of lowest energy, it predicts the thermodynamic pK_a. However, as Roszak et al. [15] discuss, various factors can influence the reaction site, including (i) the formation of a dianion, (ii) the deprotonation facilitated by pre-coordination of a base, (iii) the emergence of strained intermediates, and (iv) the enolization of ketones.

Such factors allow for the manipulation of the reaction site, resulting in the functionalization of a less acidic C–H site. In the context of unsymmetrical ketones, two acidic sites are possible. The more substituted site typically results in a more stable anion (the thermodynamic anion), while the less substituted site yields the kinetic anion. By selecting appropriate bases, synthetic chemists can

guide the reaction site to favor the kinetic or thermodynamic anion. For instance, employing lithium diisopropyl amine often produces the kinetic product, while bulkier bases like *tert*-butoxide tend to favor the thermodynamic anion. Such intricacies are absent from our basic ML model, rendering it incapable of distinguishing between different types of anions. Figure S12 shows that our model consistently predicts the lowest thermodynamic site. Therefore, users must exercise caution when relying on our model as it serves best as a guideline. Thus, the ML model should be consulted in conjunction with the expertise of a seasoned synthetic chemist.

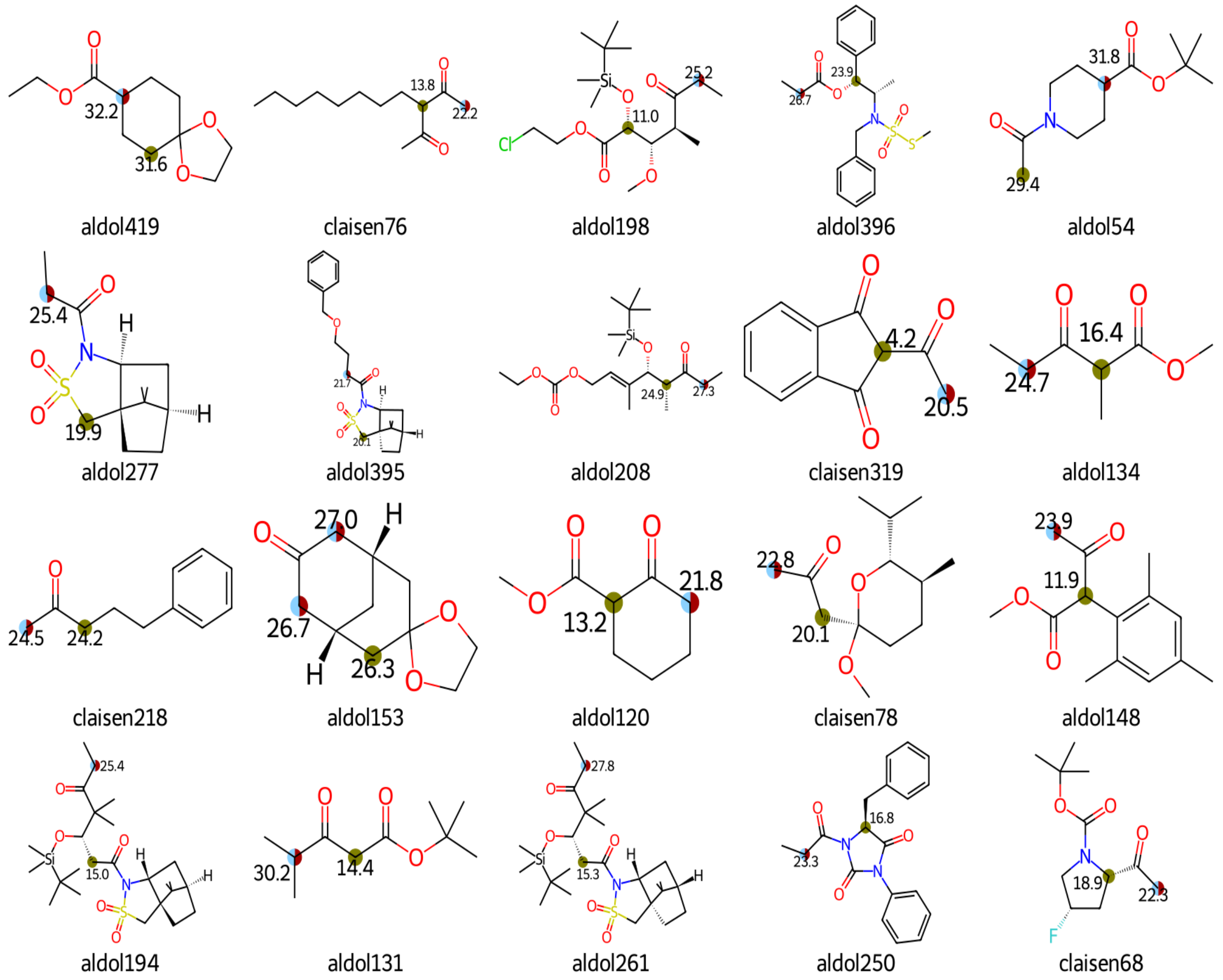

Figure S12: 20 random outliers from Reaxys: cyan highlight: reaction site; dark yellow highlight: false positive; purple highlight: false negative.

Prediction of aryl C–H borylation sites

Table S14: Data for the QM-computed pK_a values (pK_a calc) and predicted ML pK_a values (pK_a pred) for the six pharmaceutical intermediates.

compound	atom site	atom reaction	pK_a calc	pK_a pred	aromatic
1	1	0	36.7	34.7	False
	6	0	37.3	34.1	True
	7	1	34.5	40.8	True
	12	0	39.1	40.1	True
	13	0	37.8	34.8	True
2	1	0	47.2	43.4	False
	4	0	40.2	36.8	True
	9	0	37.1	34.1	True
	13	1	36.7	29.9	True
	14	0	39.2	40.8	True
	16	0	43.4	40.9	True
	17	0	46.2	41.7	True
	18	0	45.5	42.0	True
3	1	0	39.2	31.1	False
	3	0	inf	38.6	True
	7	0	41.0	31.5	True
	8	0	37.1	35.8	True
	12	0	inf	42.9	True
	13	1	46.3	44.5	True
	14	0	47.0	43.1	True
	26	0	inf	47.9	False
4	1	0	46.4	43.4	False
	4	0	42.2	38.6	True
	6	0	33.6	29.8	False

	8	0	inf	42.0	False
	9	0	inf	48.7	False
	10	0	inf	42.2	False
	13	0	42.9	28.5	True
	16	0	inf	39.5	False
	20	0	45.0	37.1	True
	21	1	39.7	33.1	True
	24	0	inf	39.4	True
	27	0	39.5	42.8	True
5	1	0	inf	43.2	False
	6	0	31.6	37.2	False
	7	0	41.7	37.4	True
	15	0	35.5	37.1	True
	16	1	40.6	38.4	True
	17	0	40.2	40.0	True
	21	0	47.4	42.4	True
	22	0	45.1	43.9	True
	24	0	inf	42.2	False
	26	0	50.4	51.2	False
6	5	0	40.3	35.6	True
	6	0	40.8	39.5	True
	10	0	22.2	39.7	False
	13	0	35.6	35.6	True
	15	0	38.7	40.2	True
	16	0	40.7	39.0	True
	17	1	34.3	33.9	True

References

1. Butina, D. *J. Chem. Inf. Comput. Sci.* **1999**, *39*, 747–750. doi:10.1021/ci9803381.
2. Neese, F. *Wiley Interdiscip. Rev.: Comput. Mol. Sci.* **2012**, *2*, 73–78. doi:10.1002/wcms.81.
3. Neese, F.; Wennmohs, F.; Becker, U.; Riplinger, C. *J. Chem. Phys.* **2020**, *152*, 224108. doi: 10.1063/5.0004608.
4. Grimme, S.; Hansen, A.; Ehlert, S.; Mewes, J.-M. *J. Chem. Phys.* **2021**, *154*, 064103. doi: 10.1063/5.0040021.
5. Marenich, A. V.; Cramer, C. J.; Truhlar, D. G. *J. Phys. Chem. B* **2009**, *113*, 6378–6396. doi:10.1021/jp810292n.
6. Yanai, T.; Tew, D. P.; Handy, N. C. *Chem. Phys. Lett.* **2004**, *393*, 51–57. doi:10.1016/j.cplett. 2004.06.011.
7. Caldeweyher, E.; Ehlert, S.; Hansen, A.; Neugebauer, H.; Spicher, S.; Bannwarth, C.; Grimme, S. *J. Chem. Phys.* **2019**, *150*, 154122. doi:10.1063/1.5090222.
8. Weigend, F.; Ahlrichs, R. *Phys. Chem. Chem. Phys.* **2005**, *7*, 3297. doi:10.1039/b508541a.
9. Rappoport, D.; Furche, F. *J. Chem. Phys.* **2010**, *133*, 134105. doi:10.1063/1.3484283.
10. Barone, V.; Cossi, M. *J. Phys. Chem. A* **1998**, *102*, 1995–2001. doi:10.1021/jp9716997.
11. Shen, K.; Fu, Y.; Li, J.-N.; Liu, L.; Guo, Q.-X. *Tetrahedron* **2007**, *63*, 1568–1576. doi:10.1016/ j.tet.2006.12.032.
12. Matthews, W. S.; Bares, J. E.; Bartmess, J. E.; Bordwell, F. G.; Cornforth, F. J.; Drucker, G. E.; Margolin, Z.; McCallum, R. J.; McCollum, G. J.; Vanier, N. R. *J. Am. Chem. Soc.* **1975**, *97*, 7006–7014. doi:10.1021/ja00857a010.
13. A. Koppel, I.; Koppel, J.; Pihl, V.; Leito, I.; Mishima, M.; M. Vlasov, V.; M. Yagupolskii, L.; Taft, R. W. *J. Chem. Soc., Perkin Trans. 2* **2000**, *0*, 1125–1133. doi:10.1039/B001792M.

14. *RDKit 2022_09_4 (Q3 2022) Release*; Zenodo, 2023. doi:10.5281/zenodo.7541264.

15. Roszak, R.; Beker, W.; Molga, K.; Grzybowski, B. A. *J. Am. Chem. Soc.* **2019**, *141*, 17142–17149. doi:10.1021/jacs.9b05895.
